# Low 6-Minute Walk Distance and Muscle Mass Predict Drop out in Cardiac Rehabilitation

**DOI:** 10.3390/healthcare8040430

**Published:** 2020-10-25

**Authors:** Ju Hyung Park, Kyu Kwon Cho, Yong Hwan Kim

**Affiliations:** 1 Department of Sports Sciences, University of Seoul, Seoul 02504, Korea; park.jh.med@gmail.com; 2 Department of Physical Education, Gangneung-Wonju National University, Gangneung 25457, Korea; kkcho@gwnu.ac.kr

**Keywords:** 6-minute walking distance, cardiac rehabilitation, dropout, muscle ratio, socioeconomic status

## Abstract

Cardiac rehabilitation after percutaneous coronary intervention decreases recurrence and mortality but has a high dropout rate. The aim of this study is to identify dropout predictors by comparing the characteristics of complete and dropout patients in cardiac rehabilitation. The study included 593 patients (455 men and 138 women) who received percutaneous coronary intervention and were enrolled in a 1-year cardiac rehabilitation program consisting of home-based cardiac rehabilitation with three center visits. Dropout was defined as participation in the first center visit but not the second or third center visits. Blood lipids, quality of life, socioeconomic status, and 6-minute walk distance measurements at the first visit were compared between participants who completed and dropped out of cardiac rehabilitation. For both men and women, the dropout rate significantly correlated with a low 6-minute walk distance and low muscle mass ratio. The dropout rate was significantly higher for men, but not women, with low education and low income. However, the dropout rate was decreased for women, but not men, with low blood pressure and triglycerides. An improved understanding of the characteristics of participants and the cardiac rehabilitation dropout rate are expected to contribute to the development of cardiac rehabilitation strategies that decrease patient dropout.

## 1. Introduction

Cardiovascular disease is a major cause of death worldwide [1]. The most common treatments for this disease include medication, percutaneous coronary intervention (PCI), and coronary artery bypass graft. Systemic healthcare, known as cardiac rehabilitation (CR), is also recommended through lifestyle modifications [2]. CR aims to lower relapse and mortality in patients with cardiovascular disease through exercise, nutrition, and psychological counseling by nurses [3]. Previous studies suggest that CR decrease cardiovascular risk factors as obesity hypertension, diabetes, dyslipidemia, as well as increase patient’s life satisfaction, by inducing positive effects on quality of life (QoL) and psychological comfort [4,5]. In addition, CR helps patients by increasing the maximum oxygen consumption and improving exercise capacity [6]. Despite the positive effects of CR, the dropout rate tends to be relatively high. In previous, 46.9% of women did not complete the CR program, and dropouts were more likely to have obesity and depression [7]. In another study, the characteristics of CR participation in 176 patients discharged from the hospital after treatment for heart disease were analyzed. There were 25 patients who completed the 18-session CR program, 47 who dropped out, and 104 who not participate at all [8]. Barriers to participation in CR include psychological problems, such as depression and anxiety and discomforts such as physical health problems. In addition, distance to the medical center, economic burden, and low physical strength can decrease participation [8,9].

Fitness and body composition have been studied as predictors of cardiovascular disease and mortality. Even among individuals with normal weight, those with low fitness have a 2.7-fold increase in cardiovascular mortality compared to those with high fitness [10]. Additionally, it has been found that the mortality rate decreases as the 6-minute walking capacity increases in people with chronic obstructive pulmonary disease [11]. Body composition studies show that high fat and low muscle mass increase the risk of cardiovascular disease [12,13]. In a study related to participation in CR, high exercise capacity and low body mass index (BMI) were found to be positive factors for participation in CR. However, studies examining factors predictive of completion of CR are rare [14]. Therefore, we hypothesized that individuals with high exercise capacity and muscle mass would have higher participation in cardiac rehabilitation. We examined the correlation of various physical variables, socioeconomic status (SES), body composition, and lipid levels with participation in CR. 

## 2. Materials and Methods 

### 2.1. Participants 

There were 650 patients who underwent PCI in our cardiology department. However, depending on the patient’s choice, there were patients who did not participate in CR or had incomplete test data. Therefore, 57 of these patients were excluded from the analysis. The criteria for inclusion in the study were participation in the CR program at least once and completion of physical fitness tests, body composition analysis, and SES surveys. A total of 593 patients (455 men and 138 women) were finally included in the analysis.

The CR program was presented by a cardiologist, and only patients who agreed to participate in the program were included. Patients who underwent PCI were instructed to participate in the CR program at 4 weeks following discharge. Patients were encouraged to participate in CR through home-based exercise for 10–12 months. A dropout patient was defined as one who did not continue with follow-up CR after the first visit. A complete patient was defined as one who continued follow-up CR for 1 year. This study complies with the Helsinki Declaration and was approved by the institutional review board (AMC 2015-0594). 

### 2.2. Cardiac Rehabilitation Program

The CR team consisted of cardiologists, exercise specialists, nutritionists, and nurses. The nurse explained to patients how to deal with precautions such as the Valsalva maneuver, how to use nitroglycerin, and how to monitor heart rate. Dietitians consulted on how to make a balanced diet, including low-sodium and low-fat diets, and menu choices when eating out. The exercise program was prescribed based on the American College of Sports Medicine guidelines [15]. Based on the results of the participant’s cardiac exercise load test, exercise intensity was set in the range of 40–70% of heart rate reserve. Patients were educated on how to exercise within the suggested range using the Rating of Perceived Exertion [16]. In addition, patients were educated to monitor their heart rate by electronic devices or radial arteries.

### 2.3. Measurements

QoL was assessed with the short Form Health Survey (SF-36), and SES was assessed through a questionnaire. Physical fitness was evaluated based on grip strength, flexibility, balance, and a 6-minute walking test (6MWT). Fasting with limited water was requested for 8 h prior to the analysis of blood lipids. The study measurements were performed in the following order: (1) questionnaire, (2) body composition and anthropometric measurements, (3) blood pressure measurement, and (4) physical fitness tests.

#### 2.3.1. Body Composition and Waist Circumference

Body weight, BMI, and muscle mass were measured using the Inbody 770 (Inbody Co., Seoul, Korea), per the manufacturer’s instructions. Waist circumference was measured twice horizontally using a tape measure at the thickest part of the navel level. If the measurements differed by more than 0.5 cm, the waist circumference was measured again. 

#### 2.3.2. Grip Strength

Grip force was measured using a Takei 5401 handle force dynamometer (TKK-5401; TAKEI Scientific Instruments Co., Ltd., Niigata, Japan). The patient stood upright with the elbows in line with the waist and chest and the hand being examined extended. The hand was placed next to the thigh, and the patient was instructed not to touch the thigh. Then, the mechanical handle was placed on the second metacarpal bone. When the patient was ready, the examiner gave the signal “go,” and the patient exerted maximum strength. The posture was not disturbed during the test [17,18]. The grip of both hands was tested twice, and the average of the highest value was calculated for each hand.

#### 2.3.3. Balance

For the balance test, the patient stood on a foot-shaped examination table, and both hands were placed on the waist. Then, the patient closed his eyes and stood on one leg. The opposite leg was bent back 90 degrees at the examiner’s “go.” The test ended when the patient opened his eyes, removed his hands from his waist, and placed both feet on the ground [17,19]. Both sides were tested twice. The average of the highest value was calculated for each leg. If the average did not exceed 2 s after starting, the test was performed again.

#### 2.3.4. 6-Minute Walk Distance (6MWD)

A 6MWD test was administered, in which the participant was allowed to walk as fast as possible for 6 min on a 60-m track. Cones were installed at both ends of the track, and the test was conducted in a safe space with a flat floor and no obstacles. The examiner started the test after the test had been fully explained and the patient understood the test method. The test started with the inspector’s “go” signal, and the patient was informed of the time in 1-minute increments. The walking pace was self-regulated, and running was prohibited [20,21]. 

#### 2.3.5. Socioeconomic Status (SES)

Socioeconomic variables were based on monthly income and education level. Household income was divided by tertiles, and the survey was conducted in Korean Won (KRW), with low (<2,000,000 KRW), middle (2,000,000–3,900,000 KRW), and high (>4,000,000 KRW) groups. The education level was also divided into three groups: low, middle school graduation; middle, high school graduation; and high, college or higher.

#### 2.3.6. Quality of Life (QoL)

A participant’s general health and QoL were measured using the Korean version of the SF-36 in a self-administered manner. In the analysis, mental, physical, and total scores were calculated using the standard calculation formula. As a result of structural equation modeling, the Comparative Fit Index was 0.984, the root mean square error of approximation was 0.039, and the standardized root mean square residual was 0.038, indicating this was a valid questionnaire [22].

### 2.4. Statistical Analysis

SPSS 25.0 (IBM SPSS Inc., Armonk, NY, USA) was used for data analysis. For complete and dropout patient characteristics, independent *t*-tests were performed to compare medical and fitness test results at the first visit. The categorical variable SES went through the process of setting it as a dummy variable. Monthly income and education level were classified as high, meddle, and low through the tertile. Comparison of complete and dropout patient was performed by chi-square test. Variables with significant differences in the comparison between complete and dropout patients were selected as the odds ratio. As a result, it was the 6MWD and muscle ratio, monthly income and education level in men, while it was the 6-MWD, muscle ratio, systolic blood pressure (SBP), and triglyceride (TG) in women. In order to convert these variables into dummy variables, a tertile was performed and classified into high, middle and low. Odds ratio was calculated by logistic regression analysis. These variables were also used as adjustment variables. The significance level was at *p* < 0.05. 

## 3. Results

### 3.1. Comparison between Complete and Dropout Patients

Table 1 shows the general characteristics of the participants. Men had significantly higher age, height, and weight than women.

Comparisons of variables measured at the initial visits of the complete and dropout groups are shown in Table 2. For men, significant differences in muscle ratio and 6MWD were observed between the dropout and complete patients. For women, significant differences in muscle ratio, 6MWD, SBP, and TG were observed between the dropout and complete patients (Table 2).

Table 3 shows the comparison between the groups according to SES. For men, there were significant differences in completing CR based on monthly income and education level. In the high-income group, 40.5% were complete patients, whereas in the low-income group, only 23.2% were complete patients (*p* = 0.016). In the high education group, 47.9% were complete patients, whereas in the middle and low education groups, only 36.7% and 15.4% were complete patients, respectively (*p* = 0.034).

### 3.2. Odds Ratio of Dropout in Cardiac Rehabilitation

Table 4 analyzes the dropout prevalence using the significant variables from Table 2 and Table 3. For men, dropout increased by 1.867 times for the low muscle ratio group as compared to the high muscle ratio group (95% confidence interval [CI] 1.059–3.635, *p* = 0.036) and 1.573 times for the low 6MWD group as compared to the high 6MWD group (95% CI 1.025–6.460, *p* = 0.044). Moreover, dropout for men increased by 2.102 times for the low income group as compared to the high income group (95% CI 1.414–3.985, *p* = 0.021) and 1.980 times for the low education group as compared to the high education group (95% CI 1.139–2.654, *p* = 0.018). For women, dropout increased by 2.538 times for the low muscle ratio group as compared to the high muscle ratio group (95% CI 1.246–6.444, *p* = 0.016) and 1.800 times for the low 6MWD group as compared to the high 6MWD group (95% CI 1.029–4.155, *p* = 0.041). In addition, the dropout for women in the low SBP and TG groups increased by 3.000 and 1.708 times, respectively, compared to the high SBP and TG groups (Table 4).

## 4. Discussion

This study was conducted with an attempt to show that exercise capacity and muscle mass influence cardiac rehabilitation adherence. The positive effects of exercise and other health behaviors for preventing and improving diabetes, obesity, and dyslipidemia and reducing the risk of cardiovascular disease are well known [23,24]. In a previous study, participation in a 12-month CR program led to a 0.74-times reduction in the death rate due to cardiovascular disease and a 0.82-times reduction in visits to the hospital emergency room [25]. However, despite these positive effects, a significant number of patients drop out of CR. To foster the development of strategies to enhance patient adherence to a CR program, we examined the fitness, anthropometry, blood lipid levels, SES, and QoL of patients who either completed or dropped out of a CR program after PCI. We compared the characteristics of the dropout and complete participants in order to identify the characteristics of people who complete CR. 

In the present study, the dropout rates were 43.1% for men and 39.9% for women; although the dropout rate of men was slightly higher than that of women, the difference was not statistically significant. Our results are similar to those from previous studies. In one study of 228 women, the dropout rate was 46.9% [7]; another study of 639 participants, comprising men and women, reported that 41.6% of participants dropped out [26]. In a relatively large Iranian cohort study of 1115 patients, 55.2% of CR participants did not complete CR [27]. Because the dropout rate varies depending on the study conditions, CR duration, and characteristics of the participants, previous researcher suggested that the CR dropout rate broadly ranged from 0% to 50% [28]. 

Although we found no significant difference in the dropout rates of men and women, other studies reported differences. In a study by Marzolini et al., significantly more women (35%) than men (29%) failed to complete 12 months of CR [29]. Conversely, there are reports that the dropout rate for women is rather low [30]. However, health experts emphasize that women’s low healthcare participation should be noted. They report that women have lower SES than men, and they have relatively low health consciousness and medical access [31,32,33]. 

A main finding of this study was that the relationship between SES and CR dropout was significant for men but not for women. According to a cross-sectional study, participation in CR was significantly reduced for patients with a low level of education, those living in rural areas rather than cities, and those with a low SES [34]. Similarly, there was a result that the CR participation rate of low-income patients was low [35]. In this study, there was no significant relationship between SES and CR for women. This may be due to a greater number of women than men with low and middle SES. In addition, the overall CR participation rate was lower for women than for men, which is consistent with the results of prior reports. For example, in a meta-analysis of men and women, CR enrollment rates were significantly lower for women (39%) than for men (45%) [36]. The reduced enrollment of women in CR is potentially influenced by the role of women as primary caretakers of children and the elderly, as well as low SES, rather than a lack of individual effort or gender differences. Therefore, there is a need for supplemental social systems to support women in enrolling in and completing CR [37,38].

Another main finding of this study is that exercise capacity, as evaluated by the 6MWT, affects the dropout rate. The 6MWT, which measures the distance that a person can walk in 6 minutes or the 6MWD, is a simple method to measure cardiopulmonary endurance without complicated equipment and is mainly used with cardiac or pulmonary patients or the elderly [21]. One study that analyzed complete and dropout CR patients found that patients with a below average 6MWD had a CR dropout rate 1.7- to 1.9-times higher than patients with an above average 6MWD [30]. This result is similar to that of this study, in which the dropout rate was 1.5-times higher in men and 1.8-times higher in women in the low 6MWD group compared to those in the high 6MWD group. These results suggest the possibility of predicting dropout, in addition to evaluating the effect of CR, based on a patient’s 6MWD.

One of the main findings of this study was that people with low muscle mass had a high CR dropout rate. While there reports that physical fitness affects adherence to cardiac rehabilitation, studies on muscle mass are very rare [14,39]. However, several studies reported that body composition had a role as a predictor of cardiovascular disease and mortality. The high-muscle and low-fat group had a lower cardiovascular hazard risk by 0.32 than that of the low-muscle and high-fat group, and the total mortality was lowered to 0.32 [13]. And another study for mortality found that fourth quartile (high muscle mass) had hazard ratios 0.80 than first quartile (low muscle mass) [40]. Muscle is typically at its maximum level when individuals are 30 years of age, and thereafter muscle decreases by 0.1% to 0.5% each year. The amount of muscle can change depending on age, strength training, and nutritional status [41]. Therefore, it is possible that people with high muscle mass had good exercise or nutritional status before the onset of heart disease. 

The SF-36 measures general health and well-being and is largely divided into sections on mental and physical health. In this study, there were no significant differences in mental health based on completion of CR. However, previous studies have suggested that psychological or mental factors such as depression and low QoL negatively affect adherence to CR [42,43]. Therefore, in future studies it may be necessary to use more specialized questionnaires that specifically evaluate issues of depression and self-satisfaction in order to gain further insight into the effect of mental factors on adherence to CR. Personal conditions such as health education, social or family support, and willingness or disposition also influence health behaviors [44,45,46]. 

The main strength of this study is that muscle mass and 6-minute walking, which are often measured for health evaluation, are expanded usability as tools to predict cardiac rehabilitation adherence. This result can be used as information to prevent dropout during cardiac rehabilitation. Because, patients with low walking capacity and muscle mass have a high CR dropout rate, various CR adherence strategies are needed for these patients. Ultimately, increasing the participation rate of cardiac rehabilitation will contribute to the prevention of heart disease recurrence and psychological relaxation. 

Nevertheless, our research has the following limitations: The individual reasons for dropout were not specifically investigated. In addition, the results of the exercise performance evaluation and the nutrition evaluation were not analyzed. Even if the patient did not come to the CR center, it cannot be excluded that the patient is engaging in healthy behavior. Additionally, this study did not assess the severity of the patient’s cardiovascular disease, and there were multiple medical staff and physical fitness testers. Therefore, the difference between therapists and examiners was an uncontrollable variable. In the future, it will be important to identify and compare relapse in complete and dropout patients. Additionally, as the elderly may have low physical activity [47], future studies should investigate other variables that may lower the CR dropout rate. Future studies should also include variables related to the use of information technology (IT). For example, some studies have reported a negative effect of IT due to cell phone addiction, whereas others have reported a positive effect of IT due to mobile healthcare programs [48,49]. It will be necessary to develop and verify the effectiveness of CR programs that promote health behaviors for cardiac patients. 

## 5. Conclusions

This study was conducted to analyze predictors affecting cardiac rehabilitation program dropout in patients undergoing PCI. People with high 6-min walking distance and muscle mass had a high rate of participation in cardiac rehabilitation. Furthermore, a low level of education and economic status was increased the dropout rate in cardiac rehabilitation in men.

## Figures and Tables

**Table 1:** Characteristics of participants.

	Men (n = 455)	Women (n = 138)	*p*
Age, years	58.7 ± 9.8	62.1 ± 9.8	<0.001 *
Height, cm	168.5 ± 27.6	154.3 ± 6.1	<0.001 *
Weight, kg	70.6 ± 10.5	59.6 ± 7.4	<0.001 *
BMI, kg/m2	25.1 ± 3.0	25.0 ± 2.8	0.744

* p < 0.05; BMI, body mass index. Data was presented mean ± standard deviation. Data analysis was used independent *t*-test.

**Table 2:** General characteristics comparison between cardiac rehabilitation dropout patients and complete patients.

	Men		Women	
	DOP	CG	DOP	CG
N (%)	196 (43.1%)	259 (56.9%)	55 (39.9%)	83 (60.1%)
Age, years	59.4 ± 9.7	57.7 ± 10.3	64.0 ± 10.9	61.0 ± 9.2
Height, cm	170.6 ± 42.6	166.0 ± 7.3	153.1 ± 6.4	154.7 ± 5.3
Weight, kg	70.6 ± 10.7	69.7 ± 9.5	58.8 ± 7.8	58.8 ± 6.4
BMI	25.0 ± 3.5	25.2 ± 2.6	25.1 ± 3.1	24.6 ± 2.2
Waist circumference, cm	90.6 ± 8.3	90.0 ± 6.6	90.9 ± 8.4	90.3 ± 6.3
Muscle mass, %	37.5 ± 7.2	40.2 ± 4.1 *	30.8 ± 6.8	35.7 ± 2.6 *
Fat, %	24.8 ± 6.3	24.6 ± 5.6	35.4 ± 6.1	33.7 ± 4.8
SBP, mmHg	120.0 ± 16.4	121.6 ± 15.2	124.2 ± 15.8	114.5 ± 14.4 *
DBP, mmHg	75.7 ± 11.5	75.4 ± 9.3	74.2 ± 10.9	74.5 ± 10.0
TC, mg/dL	138.5 ± 26.8	142.1 ± 32.7	156.2 ± 32.8	161.8 ± 46.6
TG, mg/dL	129.0 ± 69.6	120.9 ± 58.5	134.5 ± 88.1	113.7 ± 54.1 *
HDLC, mg/dL	45.3 ± 12.3	46.2 ± 9.5	49.2 ± 11.3	53.8 ± 13.1
LDLC, mg/dL	80.3 ± 24.9	81.9 ± 28.2	96.0 ± 44.6	88.9 ± 31.6
SF-36				
Physical score	1410.3 ± 375.7	1494.3 ± 309.2	1106.8 ± 463.5	1248.9 ± 303.5
Mental score	800.5 ± 255.6	834.6 ± 230	680 ± 221.9	742.2 ± 182.1
Total score	2254.5 ± 605.5	2368.6 ± 499.2	1818.6 ± 661.6	2032.8 ± 448.8
Fitness				
Grip strength, kg	35.7 ± 7.5	37.0 ± 6.7	25.9 ± 17.7	24.5 ± 6.3
Balance, seconds	7.9 ± 4.2	11.2 ± 12.6	6.0 ± 3.3	6.5 ± 7.4
6 min walking, meter	517.2 ± 99.2	554.8 ± 67.0 *	454.2 ± 92.3	490.1 ± 36.0 *

* *p* < 0.05; Data was presented mean ± standard deviation. Data analysis was used independent *t*-test. DOP, dropout patients; CG, complete group; SBP, systolic blood pressure; DBP, diastolic blood pressure; TC, total cholesterol; TG, triglyceride; HDLC, high density lipoprotein cholesterol; LDLC, low density lipoprotein cholesterol.

**Table 3:** Socioeconomic status comparison between cardiac rehabilitation dropout patients and complete patients.

	DOP (n = 196)	CG (n = 259)	*p*	DOP (n = 55)	CG (n = 83)	*p*
Monthly income ^a^						
High	55(28.1%)	105(40.5%)	0.016 *	14(25.5%)	20(24.1%)	0.584
Middle	68(34.7%)	94(36.3%)		19(34.5%)	27(32.5%)	
Low	70(37.2%)	60(23.2%)		22(40.0%)	36(43.4%)	
Education level ^b^						
High	64(32.7%)	124(47.9%)	0.034 *	11(20.0%)	23(27.7%)	0.863
Middle	98(50.0%)	95(36.7%)		16(29.1%)	20(24.1%)	
Low	34(17.3%)	40(15.4%)		28(50.9%)	40(48.2%)	

* *p* < 0.05; Data was presented number of patients (%); Data analysis was used chi-square test; DOP, dropout patients; CG, complete group. ^a^ *Monthly income:* low (<2,000,000 KRW); middle (2,000,000–3,900,000 KRW); and high (>4,000,000 KRW). ^b^ *Education level:* low (to middle school graduation); middle (to high school graduation); and high (to college or higher).

**Table 4:** Adjusted odds ratios of cardiac rehabilitation dropout.

	Classification	Values	Adjusted OR (95% CI)	*p*
Men				
Muscle mass%	High	≥41.7	Reference	-
	Middle	41.6–38.2	1.604(0.814–3.160)	0.172
	Low	≤38.3	1.867(1.059–3.635)	0.036 *
6 min walking, meter	High	≥572.5	Reference	-
	Middle	507.4–572.4	1.031(0.786–5.249)	0.124
	Low	≤507.5	1.573(1.025–6.460)	0.044 *
Monthly income	High	>4,000,000	Reference	-
	Middle	2,000,000–3,900,000	1.534(0.954–1.925)	0.241
	Low	<2,000,000	2.102(1.414–3.985)	0.021*
Education	High	Above college	Reference	-
	Middle	To high school	1.426(0.724–2.902)	0.219
	Low	To middle school	1.980(1.136–2.654)	0.018 *
Women				
Muscle mass%	High	≥35.7	Reference	-
	Middle	31.3–35.6	1.800(1.005–5.692)	0.019 *
	Low	≤31.4	2.538(1.246–6.444)	0.016 *
6 min walking, meter	High	≥504.3	Reference	-
	Middle	432.3–504.2	1.011(0.904–4.012)	0.190
	Low	≤432.4	1.800(1.029–4.155)	0.041 *
SBP, mmHg	Low	≤111.0	Reference	-
	Middle	111.1–125.1	1.543(0.464–5.133)	0.480
	High	≥125.2	3.000(1.036–9.616)	0.045 *
TG, mg/dl	Low	≤93.4	Reference	-
	Middle	93.4–131.6	1.555(0.471–5.133)	0.469
	High	≥131.7	1.708(1.022–5.585)	0.036 *

* *p* < 0.05; Data analysis was used logistic regression; 6MWD, 6-minute walk distance; SBP, systolic blood pressure; TC, total cholesterol; TG, triglyceride.

## References

[1] Abubakar I., Tillmann T., Banerjee A. (2015). Global, regional, and national age-sex specific all-cause and cause-specific mortality for 240 causes of death, 1990–2013: A systematic analysis for the Global Burden of Disease Study 2013. Lancet.

[2] Kachur S., Chongthammakun V., Lavie C.J., De Schutter A., Arena R., Milani R.V., Franklin B.A. (2017). Impact of cardiac rehabilitation and exercise training programs in coronary heart disease. Prog. Cardiovasc. Dis..

[3] Fletcher G., Ades P., Kligfield P., Arena R., Balady G., Bittner V., Coke L., Fleg J., Forman D., Gerber T. (2013). Exercise standards for testing and training: A scientific statement from the American Heart Association. Circulation.

[4] Piepoli M.F., Corra U., Benzer W., Bjarnason-Wehrens B., Dendale P., Gaita D., McGee H., Mendes M., Niebauer J., Zwisler A.-D.O. (2010). Secondary prevention through cardiac rehabilitation: From knowledge to implementation. A position paper from the Cardiac Rehabilitation Section of the European Association of Cardiovascular Prevention and Rehabilitation. Eur. J. Cardiovasc. Prev. Rehabil..

[5] Kim S.G., Choi S.B., Kim Y.H. (2019). Effect of short-term cardiac rehabilitation on quality of life according to socioeconomic status. J. Men’s Health.

[6] Chen Y.-W., Wang C.-Y., Lai Y.-H., Liao Y.-C., Wen Y.-K., Chang S.-T., Huang J.-L., Wu T.-J. (2018). Home-based cardiac rehabilitation improves quality of life, aerobic capacity, and readmission rates in patients with chronic heart failure. Medicine.

[7] Sanderson B.K., Bittner V. (2005). Women in cardiac rehabilitation: Outcomes and identifying risk for dropout. Am. Heart J..

[8] Kim C., Lim H.S., Ahn J.K., Bang I.K., Lee S.M., Kim Y.J. (2002). The reasons that cardiac patients did not participate in and drop out from the cardiac rehabilitation program. J. Korean Acad. Rehabil. Med..

[9] Turk-Adawi K., Sarrafzadegan N., Grace S.L. (2014). Global availability of cardiac rehabilitation. Nat. Rev. Cardiol..

[10] Church T.S., LaMonte M.J., Barlow C.E., Blair S.N. (2005). Cardiorespiratory fitness and body mass index as predictors of cardiovascular disease mortality among men with diabetes. Arch. Intern. Med..

[11] Pinto-Plata V., Cote C., Cabral H., Taylor J., Celli B. (2004). The 6-min walk distance: Change over time and value as a predictor of survival in severe COPD. Eur. Respir. J..

[12] Stevens J., Cai J., Evenson K.R., Thomas R. (2002). Fitness and fatness as predictors of mortality from all causes and from cardiovascular disease in men and women in the lipid research clinics study. Am. J. Epidemiol..

[13] Srikanthan P., Horwich T.B., Tseng C.H. (2016). Relation of muscle mass and fat mass to cardiovascular disease mortality. Am. J. Cardiol..

[14] Jackson L., Leclerc J., Erskine Y., Linden W. (2005). Getting the most out of cardiac rehabilitation: A review of referral and adherence predictors. Heart.

[15] ACSM, American College Sports Medicine (2017). ACSM’s Guidelines for EXERCISE Testing and Prescription.

[16] American Association of Cardiovascular and Pulmonary Rehabilitation (AACVPR) (2013). Guidelines for Cardia Rehabilitation and Secondary Prevention Programs-(with Web Resource).

[17] ACSM (2013). ACSM’s Health-Related Physical Fitness Assessment Manual.

[18] Maurissen J.P., Marable B.R., Andrus A.K., Stebbins K.E. (2003). Factors affecting grip strength testing. Neurotoxicology Teratol..

[19] Michikawa T., Nishiwaki Y., Takebayashi T., Toyama Y. (2009). One-leg standing test for elderly populations. J. Orthop. Sci..

[20] Rikli R.E., Jones C.J. (2013). Senior Fitness Test Manual.

[21] Enright P.L. (2003). The six-minute walk test. Respir. Care.

[22] Han C.W., Lee E.J., Sekita Y., Kohzuki M. (2009). Use of structural equation modeling to test construct validity of the SF-36 health survey among community-dwelling Elderly in Korea. Korea Care Manag. Res..

[23] Ko D.H., Lee K.H., Kim Y.H. (2020). Longitudinal study on the relative risk of type 2 diabetes mellitus according to obesity and physical activity. J. Men’s Health.

[24] Kim S. (2020). Physical activity and cardiorespiratory fitness attenuate the impact of sarcopenic-obesity on cardiovascular disease risk in Korean men. J. Men’s Health.

[25] Anderson L., Oldridge N., Thompson D.R., Zwisler A.-D., Rees K., Martin N., Taylor R.S. (2016). Exercise-based cardiac rehabilitation for coronary heart disease: Cochrane systematic review and meta-analysis. J. Am. Coll. Cardiol..

[26] Andrew G.M., Oldridge N.B., Parker J.O., Cunningham D.A., Rechnitzer P.A., Jones N.L., Buck C., Kavanagh T., Shephard R., Sutton J.R. (1981). Reasons for dropout from exercise programs in post-coronary patients. Med. Sci. Sports Exerc..

[27] Sarrafzadegan N., Rabiei K., Shirani S., Kabir A., Mohammadifard N., Roohafza H. (2007). Drop-out predictors in cardiac rehabilitation programmes and the impact of sex differences among coronary heart disease patients in an Iranian sample: A cohort study. Clin. Rehabil..

[28] Karmali K.N., Davies P., Taylor F., Beswick A., Martin N., Ebrahim S. (2014). Promoting patient uptake and adherence in cardiac rehabilitation. Cochrane Database Syst. Rev..

[29] Marzolini S., Brooks D., Oh P.I. (2008). Sex differences in completion of a 12-month cardiac rehabilitation programme: An analysis of 5922 women and men. Eur. J. Cardiovasc. Prev. Rehabil..

[30] Sanderson B.K., Phillips M.M., Gerald L., DiLillo V., Bittner V. (2003). Factors associated with the failure of patients to complete cardiac rehabilitation for medical and nonmedical reasons. J. Cardiopulm. Rehabil. Prev..

[31] Lopez-Gonzalez A.A., Bennasar-Veny M., Tauler P., Aguilo A., Tomas-Salva M., Yanez A. (2015). Socioeconomic inequalities and age and gender differences in cardiovascular risk factors. Gac. Sanit..

[32] Clarke K.W., Gray D., Keating N.A., Hampton J.R. (1994). Do women with acute myocardial infarction receive the same treatment as men?. BMJ.

[33] Mosca L., Barrett-Connor E., Kass Wenger N. (2011). Sex/gender differences in cardiovascular disease prevention: What a difference a decade makes. Circulation.

[34] Shanmugasegaram S., Oh P., Reid R.D., McCumber T., Grace S.L. (2013). Cardiac rehabilitation barriers by rurality and socioeconomic status: A cross-sectional study. Int. J. Equity Health.

[35] Suaya J.A., Shepard D.S., Normand S.-L.T., Ades P.A., Prottas J., Stason W.B. (2007). Clinical perspective. Circulation.

[36] Samayoa L., Grace S.L., Gravely S., Scott L.B., Marzolini S., Colella T.J. (2014). Sex differences in cardiac rehabilitation enrollment: A meta-analysis. Can. J. Cardiol..

[37] Song M.Y., Lim W.Y., Kim J.I. (2015). Gender based health inequality and impacting factors. Korean J. Women Health Nurs..

[38] Song L., Lin N. (2009). Social capital and health inequality: Evidence from Taiwan. J. Health Soc. Behav..

[39] Moore S.M., Dolansky M.A., Ruland C.M., Pashkow F.J., Blackburn G.G. (2003). Predictors of women’s exercise maintenance after cardiac rehabilitation. J. Cardiopulm. Rehabil. Prev..

[40] Srikanthan P., Karlamangla A.S. (2014). Muscle mass index as a predictor of longevity in older adults. Am. J. Med..

[41] Curcio F., Ferro G., Basile C., Liguori I., Parrella P., Pirozzi F., Della-Morte D., Gargiulo G., Testa G., Tocchetti C.G. (2016). Biomarkers in sarcopenia: A multifactorial approach. Exp. Gerontol..

[42] Glazer K.M., Emery C.F., Frid D.J., Banyasz R.E. (2002). Psychological predictors of adherence and outcomes among patients in cardiac rehabilitation. J. Cardiopulm. Rehabil. Prev..

[43] Oldridge N., Gottlieb M., Guyatt G., Jones N., Streiner D., Feeny D. (1998). Predictors of health-related quality of life with cardiac rehabilitation after acute myocardial infarction. J. Cardiopulm. Rehabil. Prev..

[44] Baranowski T., Perry C.L., Parcel G.S. (2002). How individuals, environments, and health behavior interact. Health Behav. Health Educ. Theory Res. Pract..

[45] Umberson D., Crosnoe R., Reczek C. (2010). Social relationships and health behavior across the life course. Annu. Rev. Sociol..

[46] Allen J., Markovitz J., Jacobs Jr D.R., Knox S.S. (2001). Social support and health behavior in hostile black and white men and women in CARDIA. Psychosom. Med..

[47] Kong S., So W.-Y. (2017). Gender differences in body composition, physical activity level, physical fitness, and bone mineral density among elderly individuals living alone compared to those living with their spouses. J. Men’s Health.

[48] Kim H.-S., Lee K.-H., Kim H., Kim J.H. (2014). Using mobile phones in healthcare management for the elderly. Maturitas.

[49] Qian Y., Zhang L., Zheng X., Wang H., Zhou Y., Sun J. (2020). Cell phone addiction and apps activities among Chinese medical students: Prevalence and risk factors. J. Men’s Health.

